# Early calcium increase triggers the formation of olfactory long-term memory in honeybees

**DOI:** 10.1186/1741-7007-7-30

**Published:** 2009-06-16

**Authors:** Emmanuel Perisse, Valérie Raymond-Delpech, Isabelle Néant, Yukihisa Matsumoto, Catherine Leclerc, Marc Moreau, Jean-Christophe Sandoz

**Affiliations:** 1 Centre de Recherches sur la Cognition Animale (CRCA), Université de Toulouse, CNRS, Toulouse, France; 2 Centre de Biologie de Développement (CBD), Université de Toulouse, CNRS, Toulouse, France; 3 GDR 2688 'Role of calcium in gene expression in normal and pathological conditions'

## Abstract

**Background:**

Synaptic plasticity associated with an important wave of gene transcription and protein synthesis underlies long-term memory processes. Calcium (Ca^2+^) plays an important role in a variety of neuronal functions and indirect evidence suggests that it may be involved in synaptic plasticity and in the regulation of gene expression correlated to long-term memory formation. The aim of this study was to determine whether Ca^2+ ^is necessary and sufficient for inducing long-term memory formation. A suitable model to address this question is the Pavlovian appetitive conditioning of the proboscis extension reflex in the honeybee *Apis mellifera, *in which animals learn to associate an odor with a sucrose reward.

**Results:**

By modulating the intracellular Ca^2+ ^concentration ([Ca^2+^]i) in the brain, we show that: (i) blocking [Ca^2+^]i increase during multiple-trial conditioning selectively impairs long-term memory performance; (ii) conversely, increasing [Ca^2+^]i during single-trial conditioning triggers long-term memory formation; and finally, (iii) as was the case for long-term memory produced by multiple-trial conditioning, enhancement of long-term memory performance induced by a [Ca^2+^]i increase depends on *de novo *protein synthesis.

**Conclusion:**

Altogether our data suggest that during olfactory conditioning Ca^2+ ^is both a necessary and a sufficient signal for the formation of protein-dependent long-term memory. Ca^2+ ^therefore appears to act as a switch between short- and long-term storage of learned information.

## Background

Activity-dependent modifications of synaptic strength are thought to form a basis for the neuronal changes that are associated with the formation of long-term memory (LTM) [1,2]. Intensive previous work has sought to unravel the molecular cascades involved in LTM formation in different animal models from vertebrates to invertebrates [3-5]. The hallmark of LTM is that it requires an important wave of protein synthesis [6,7]. The formation of LTM was also shown to depend on a number of molecular actors, such as adenylate cyclase (AC), calcium/calmodulin-dependent kinase II (Ca^2+^/CaMKII), cyclic adenosine monophosphate (cAMP) responsive element binding protein (CREB) factor, calcineurin and nitric oxide (NO) among others [4,8-14]. Usually, LTM is formed after multiple learning trials, so that trial repetition is thought to induce specific molecular cascades leading to the gene expression and protein synthesis necessary for LTM formation. Many efforts have been invested in finding the molecular trigger at the start of these cascades, which may thus represent the earliest event in LTM formation. However, until now, this search has remained rather inconclusive.

One possible candidate for this role is Ca^2+^. Indeed, most molecules shown to be involved in LTM formation depend directly or indirectly on Ca^2+^, usually through Ca^2+^-binding proteins. A highly studied example and one of the earliest actors in these cascades is AC [9,12,13,15,16]. Several studies have shown that the AC subtypes involved in LTM are activated by the Ca^2+^-binding protein calmodulin (CaM) [15,17,18] and consequently suggest that Ca^2+^, and not AC, may be the initial trigger for LTM formation. Another argument for the central role that Ca^2+ ^may play in LTM formation is the demonstration that Ca^2+ ^is crucial for the establishment of long-term potentiation, a well-studied cellular model thought to underlie LTM [19,20]. Several previous studies have made a link between Ca^2+ ^levels and memory processing [21-25]. In these studies however, pharmacological drugs used *in vivo *and *in vitro *acted on different receptors involved in the modulation of intracellular Ca^2+ ^concentration ([Ca^2+^]i) but did not act directly on Ca^2+^. Moreover, most studies suggested that Ca^2+ ^is necessary for LTM formation, but they did not show that it may be sufficient; that is, that in association with a learning procedure producing only short-term memory, Ca^2+ ^may induce LTM formation. The aim of our work is to demonstrate the direct role of Ca^2+ ^in memory formation and to show that it is both necessary and sufficient during conditioning to induce LTM formation. Our hypothesis is that an increase in the [Ca^2+^]i during multiple-trial learning could be a key for triggering long-lasting gene expression-dependent phenomena involved in LTM formation.

The honeybee *Apis mellifera *is a well-established model to study the molecular basis of memory formation [26,27]. It presents important learning and memory capacities [27] and a brain accessible to different neurophysiological techniques, and genomic analysis is now possible since its genome has been sequenced [28].

To investigate whether Ca^2+ ^is necessary and sufficient for LTM formation, we have used the Pavlovian appetitive conditioning of the proboscis extension reflex (PER) [29], in which bees learn to associate an odor conditioned stimulus (CS) with a sucrose unconditioned stimulus (US). In this assay, several memory phases have been described. In particular, a single conditioning trial leads only to short and mid-term memories, lasting about one day. However, multiple conditioning trials spaced by 10 minutes specifically lead to *de novo *protein synthesis-dependent LTM, lasting 72 hours or more [30]. Shortly after such training, an increase of Ca^2+ ^responses was found in olfactory brain structures [31,32]. According to our hypothesis Ca^2+ ^could be the primary trigger of LTM after multiple conditioning trials. Using pharmacological and caged compounds approaches to modify [Ca^2+^]i during learning, we demonstrate that Ca^2+ ^is both necessary and sufficient during acquisition for LTM formation.

## Results

### Inhibition of [Ca^2+^]i increase during learning specifically impairs long-term memory formation

**Figure 1:**
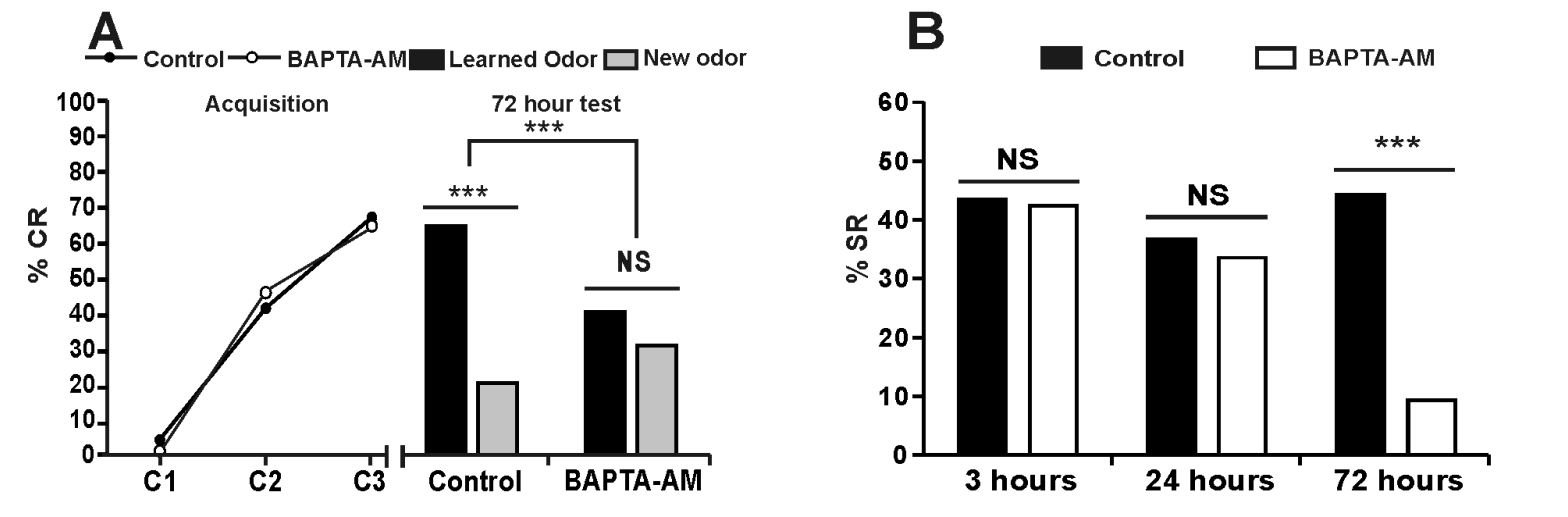
**Inhibition of [Ca^2+^]i increase using BAPTA-AM blocks long-term memory formation**. Acquisition and retention performances following an injection of BAPTA-AM 500 μM, an intracellular Ca^2+ ^chelator, or saline 1 h before training. **A. **Percentages of conditioned responses (%CR) increase during the three conditioning trials (C1, C2 and C3) for the control group (*n *= 70, *Q *= 60.0, *P *< 0.001) and for the BAPTA-AM group (*n *= 85, *Q *= 81.0, *P *< 0.001), without any difference between groups (*U *= 278.5, *P *= 0.93). Response to the conditioned stimulus (CS) after 72 h was significantly lower in the BAPTA-AM than in the control group (χ^2 ^= 9.1, *P *= 0.0026). Moreover, bees responded significantly more to the CS than to the new odor in the control group (χ^2 ^= 24.3, *P *< 0.001) but not in the BAPTA-AM group (χ^2 ^= 2.0, *P *= 0.15). Consequently, specific response proportion (% individuals responding to the CS and not to the new odor) was significantly lower for BAPTA-AM than for control bees (χ^2 ^= 15.55, *P *< 0.001). Furthermore, control and treated animals responded similarly to the new odor (χ^2 ^= 2.25, *P *= 0.13). **B. **The percentage of specific response (% SR) at 3 h (Control: *n *= 125; BAPTA-AM: *n *= 120) and 24 h (Control: *n *= 112; BAPTA-AM: *n *= 117) were not affected by treatment with BAPTA-AM (respectively: χ^2 ^= 0.65, *P *= 0.42 and χ^2 ^= 0.43, *P *= 0.51). The % SR presented at 72 h corresponds to the data of Figure 1A. (***: *P *< 0.001, NS: non-significant).

Three conditioning trials with 10-min inter-trial intervals normally lead to high LTM performance at 72 h. To test whether Ca^2+ ^is necessary for LTM formation, we first evaluated the effect of 1,2-bis-(o-aminophenoxy)-ethane-N,N,N',N'-tetraacetic acid, tetraacetoxymethyl ester (BAPTA-AM), a membrane permeant Ca^2+ ^chelator, injected 1 h before conditioning on memory performance at 72 h. The results in Figure 1A (left panel) show that the percentage of conditioned responses (%CR) increased similarly during conditioning in BAPTA-treated and control animals, indicating that BAPTA treatment does not affect acquisition performance. However, as shown on the histogram of Figure 1A (right panel, black bars), after 72 h the responses to the learned odor (CS) were significantly reduced in BAPTA-treated animals, relative to controls. To check whether memory was specific to the learned odor, we systematically compared performance to the CS and to a new odor in the two groups (Figure 1A, grey bars right panel). After 72 h, while control animals responded significantly less to a new odor than to the CS, BAPTA-treated animals responded similarly to both stimuli. Furthermore, control and BAPTA-treated animals responded similarly to the new odor. All these data indicate that control animals have strong CS-specific LTM while BAPTA-treated animals present no such specific LTM.

Comparison of CS-specific responses (SR) (% SR, corresponding to the proportion of individuals responding to the CS but not to the new odor) between groups confirmed the significant reduction of LTM performance after treatment with BAPTA-AM, relative to controls (Figure 1B). We also checked that memory performances at earlier retention times corresponding to earlier memory phases were not affected by treatment with BAPTA-AM. At 3 h and at 24 h, SR were high and similar in treated and control groups. In addition, there was no effect on LTM performance when BAPTA-AM was injected 1 h after conditioning (Additional file 1). Thus in accordance with our hypothesis, a treatment that inhibits [Ca^2+^]i increase during conditioning selectively impaired LTM performance, indicating that Ca^2+ ^increase during learning is necessary for LTM formation.

### Increase of [Ca^2+^]i specifically enhances long-term retention

**Figure 2:**
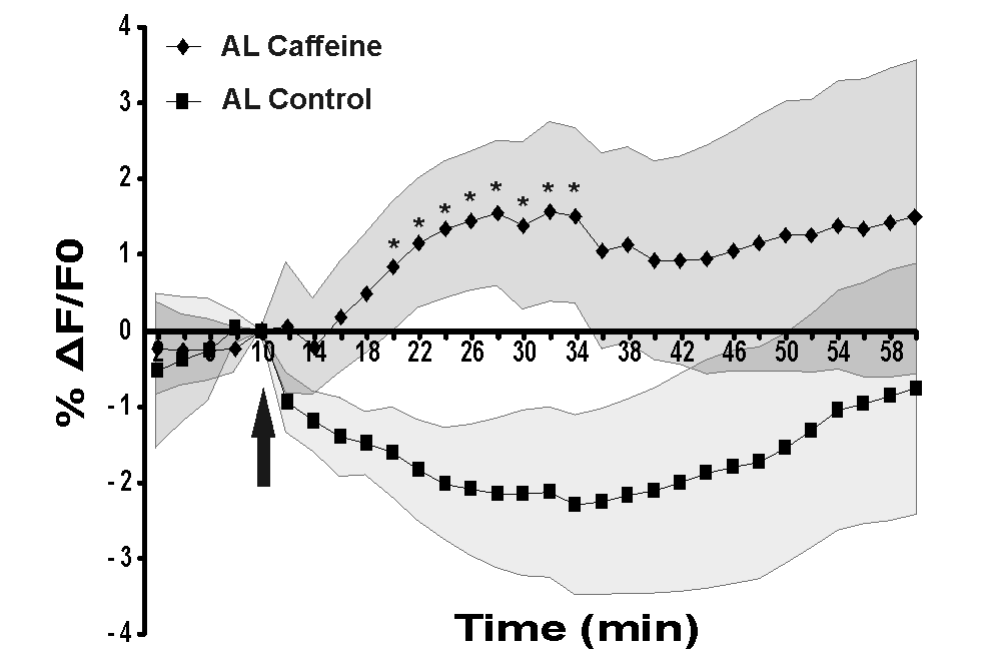
**Caffeine induces a transient Ca^2+^increase in the honeybee brain**. Relative fluorescence changes (% ΔF/F0) in the antennal lobe (AL) after an application of 10 μl of 20 mM caffeine (*n *= 10) or saline (*n *= 7) 10 min after the start of the recording (black arrow) and during 50 minutes. Between 10 and 24 min after caffeine application, the % ΔF/F0 recorded for the caffeine animals is significantly higher than for the control animals. (*: *P *< 0.05, *t*-test). Note that the drop in fluorescence observed in the saline recording is due to a change in the volume of solution between brain and objective.

The previous experiment shows that Ca^2+ ^is necessary to establish LTM, but is it sufficient? To answer this question we increased [Ca^2+^]i during one-trial conditioning, a training protocol normally inefficient to produce LTM [27]. Caffeine, which induces the release of Ca^2+ ^from ryanodine-sensitive stores [33], was used to increase [Ca^2+^]i in the honeybee brain. As a prerequisite for this experiment, we confirmed with Ca^2+ ^imaging experiments that an injection of caffeine (20 mM) induces an increase of [Ca^2+^]i. The Ca^2+ ^signal was recorded during 60 min in three different olfactory structures (antennal lobe, alpha lobe of the mushroom bodies and lateral protocerebrum). After 10 min of recording, caffeine solution or saline was injected. As shown for the antennal lobe (Figure 2), and also for the two other recorded structures, caffeine application significantly increased [Ca^2+^]i during a 14-min time window, compared with saline application.

**Figure 3:**
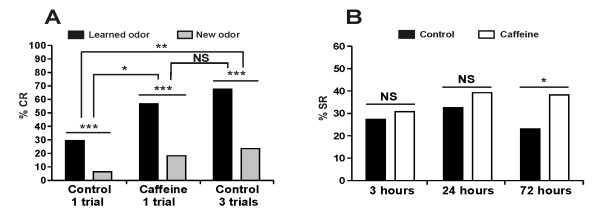
**Increase of [Ca^2+^]i using caffeine triggers long-term memory formation**. Retention performances following an injection of caffeine (20 mM) or saline solution 20 min before one- or three-trial conditioning. **A. **Conditioned response (CR) to the learned odor at 72 h was significantly higher for the caffeine group (*n *= 60) than for the one-trial conditioning group (*n *= 78) (χ^2 ^= 10.3, *P *= 0.0013), but not different from the three-trial conditioning group (*n *= 68) (χ^2 ^= 1.64, *P *= 0.2). However, the response to the new odor was significantly different between the caffeine group and the one-trial conditioning (χ^2 ^= 4.7, *P *= 0.03). Nevertheless, the caffeine group responded in the same way to the new odor than the three-trial conditioning group (χ^2 ^= 3.2, *P *= 0.07). In addition, the control one-trial conditioning, the caffeine group and the control three-trial conditioning responded significantly more to the learned odor than to the new odor (respectively: McNemar χ^2 ^= 14.4, *P *< 0.001; McNemar χ^2 ^= 19.3, *P *< 0.001; McNemar χ^2 ^= 23.3, *P *< 0.001). Overall, specific response (SR) proportion of the caffeine group was significantly increased compared with those of the control one-trial conditioning (χ^2 ^= 3.9, *P *= 0.049) and was not different from the control three-trial conditioning (χ^2 ^= 0.9, *P *= 0.33). **B. **The percentage of specific responses (% SR) for caffeine and for the control one-trial conditioning at 3 h (Control: *n *= 95; Caffeine: *n *= 78) and at 24 h (Control: *n *= 77; Caffeine: *n *= 84) were not affected by caffeine treatment (respectively: χ^2 ^= 0.2, *P *= 0.62; χ^2 ^= 0.8, *P *= 0.37). The % SR presented at 72 h corresponds to the data of Figure 3A (*: *P *< 0.05, **: *P *< 0.01, ***: *P *< 0.001, NS: non-significant).

For behavioral experiments, caffeine was injected 20 min prior to one-trial conditioning. The caffeine-elicited Ca^2+ ^release during one-trial conditioning induced a strong increase in responses to the CS at 72 h, relative to saline injection (Figure 3A). This increased response reached a similar level to that obtained after three-trial conditioning. All groups responded significantly more to the learned odor than to the new odor. However, a significant response increase was also observed to the new odor in the caffeine group compared with the one-trial conditioning group. Such an increase is not surprising as there is some behavioral generalization from the learned odor to the new odor, and increasing memory for the learned odor through caffeine treatment may increase generalization responses to the novel odor [34]. As shown in Figure 3B at 72 h, caffeine treatment increased olfactory memory as the percentage of SR (% SR) was significantly increased relative to one-trial conditioning control. Caffeine treatment had to be associated with a conditioning trial, as caffeine injected 20 min before a CS-only presentation did not lead to any LTM performance (Additional file 2).

In order to establish whether this increase affects memory before 72 h, we compared the SR of caffeine-treated and control one-trial conditioning animals at 3 h and at 24 h. In both cases, the CS-SR were similar, showing that the effect of caffeine is specific to LTM formation. It should be noted that the drop in % SR of control animals at 72 h represents natural memory decay for one-trial conditioning. Thus, the [Ca^2+^]i increase during one-trial conditioning induces LTM formation. A similar but only near-significant effect was observed on LTM performance when caffeine was injected immediately after one-trial conditioning. However, caffeine treatment had no effect when applied 1 h after one-trial conditioning (Additional file 1).

**Figure 4:**
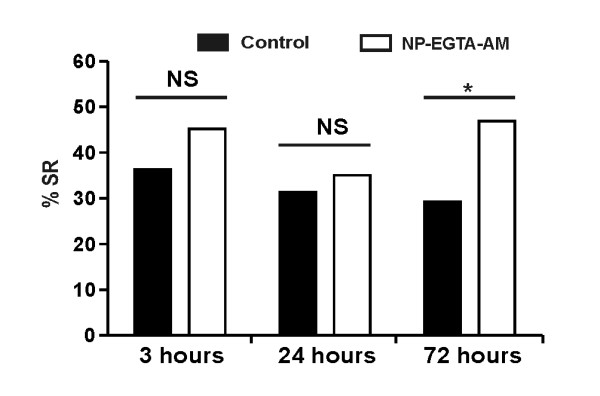
**Increase of [Ca^2+^]i by uncaging of NP-EGTA-AM triggers long-term memory formation**. Retention performances, as the percentage of specific response (% SR, % individuals responding to the conditioned stimulus and not to the new odor), following an injection of NP-EGTA-AM (20 mM) or saline solution 1 h before one-trial conditioning. The SR of the NP-EGTA-AM (*n *= 64) group was significantly higher than the control (*n *= 72) at 72 h (χ^2 ^= 4.53, *P *= 0.033). However, at 3 h (Control: *n *= 66; NP-EGTA-AM: *n *= 65) and at 24 h (Control: *n *= 102; NP-EGTA-AM: *n *= 111) the SR were no significantly different (respectively: χ^2 ^= 1.1, *P *= 0.30; χ^2 ^= 0.3, *P *= 0.56). (*: *P *< 0.05, NS: non-significant).

As caffeine has broader effects than just acting on Ca^2+ ^levels [35-37], a specific intracellular Ca^2+ ^donor, the caged *O*-Nitrophenyl-ethyleneglycol-bis(β-aminoethyl)-N,N,N',N'-tetraacetoxymethyl ester (NP-EGTA-AM) was used to confirm the promnesic effect of Ca^2+^. As shown in Figure 4, Ca^2+ ^release by uncaging of NP-EGTA-AM 5 min before one-trial conditioning also induced a specific LTM increase at 72 h. This increase was not found at 3 h or at 24 h. Altogether, these data indicate that increasing [Ca^2+^]i during one-trial conditioning leads to a specific olfactory LTM enhancement. Therefore, associated with one-trial, Ca^2+ ^is sufficient for LTM formation.

### Long-term memory formation induced by an increase of [Ca^2+^]i depends on protein synthesis

**Figure 5:**
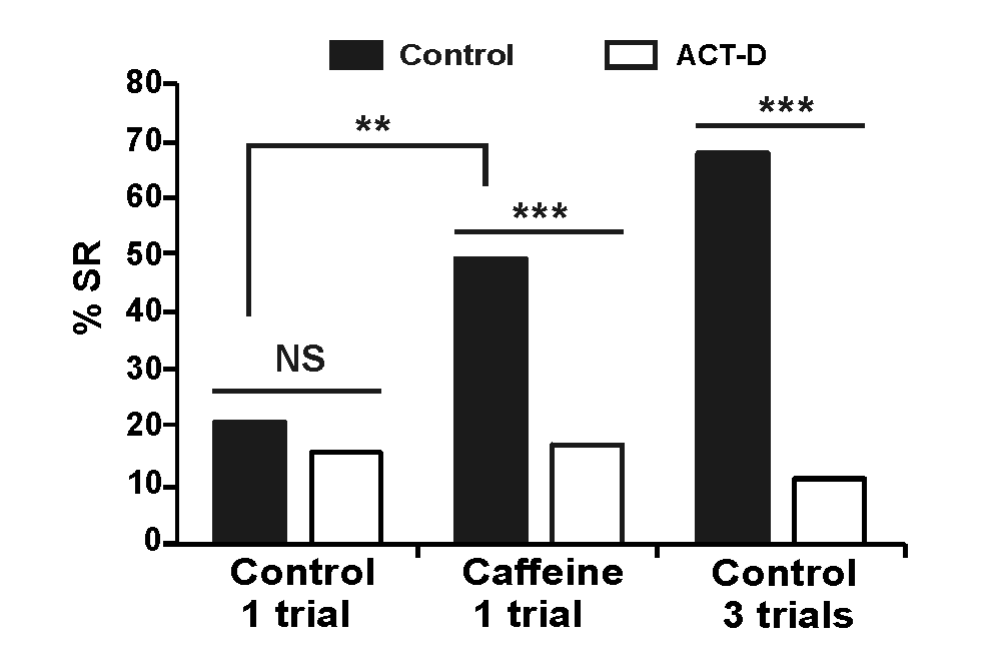
**Long-term memory increase induced by Ca^2+ ^is dependent on *de novo *gene transcription**. Control bees are compared with bees that received an injection of actinomycin-D (ACT-D) 3 h after conditioning. In control bees (black bars), injection of caffeine 20 min before one-trial conditioning induced a significant increase in retention performance (percentage of specific response, % SR, % individuals responding to the conditioned stimulus and not to the new odor) at 72 h, compared with bees which received a one-trial conditioning (χ^2 ^= 10.41, *P *= 0.0013). This replicates the results shown in Figure 3. Bees which received three conditioning trials showed high long-term memory (LTM) performance, as usual. Injection of ACT-D (white bars) almost totally erased the promnesic effect of caffeine, so that there was no longer any difference between one-trial and one-trial plus caffeine bees. Bees of the caffeine group injected with ACT-D (*n *= 62) thus showed a significant decrease of SR compared with control bees (*n *= 61) (χ^2 ^= 16.62, *P *< 0.001). No difference appears for the control one trial conditioning (χ^2 ^= 0.24, *P *= 0.62; Control: *n *= 64; ACT-D: *n *= 69). LTM produced by three-trial conditioning was also utterly erased (χ^2 ^= 40.29, *P *< 0.001; control: *n *= 63; ACT-D: *n *= 70).

LTM is dependent on a new wave of protein synthesis, required for the functional and structural synaptic modifications involved in long-term storage of information [6,30]. To test whether the LTM formed after an increase in [Ca^2+^]i is dependent upon protein synthesis, we replicated the previous experiment in the presence of the transcription inhibitor, actinomycin-D (ACT-D). Bees were subjected to one-trial conditioning associated with the injection of caffeine (20 mM) or saline solution, followed 3 h later either by an injection of ACT-D (experimental group) or saline solution (control group). These groups were then tested at 72 h. As shown in Figure 5, performance in the one-trial group injected with caffeine was significantly higher than in the one-trial control group, reproducing the previous induction of LTM by caffeine. This increase of SR performance was totally erased in the corresponding experimental group injected with ACT-D, so that performance was equivalent to that in the one-trial control group. We also performed a control experiment with three-trial conditioning which received or not ACT-D. Performances of this control three-trials conditioning group were strongly affected by ACT-D injection, as already described in the literature [38]. Thus, the increase in LTM performances in the caffeine-injected one-trial group is associated with *de novo *protein synthesis during consolidation.

## Discussion

Here we show that: (i) treatment that inhibits [Ca^2+^]i increase during multiple-trial conditioning selectively impairs LTM performance; (ii) conversely, treatments that increase [Ca^2+^]i during one-trial conditioning greatly enhance LTM performance; and finally, (iii) as was the case for LTM produced by multiple-trial conditioning, the increased memory performance at long-term induced by an increase of [Ca^2+^]i depends on *de novo *protein synthesis. Altogether our data suggest that Ca^2+ ^is both a necessary and a sufficient signal during olfactory conditioning for the formation of protein-dependent LTM.

The repetition of associative CS-US trials is thought to be the key for the formation of an odor-specific protein-dependent LTM. Therefore, additional conditioning trials act as confirmations of the information gained during the first conditioning trial. A question that was left unanswered until now was which intracellular messengers were keeping track of the first associative trial? Our present results suggest that Ca^2+ ^could play this role, priming neuronal units for subsequent trials and preparing for LTM formation. Indeed, we show that multiple trials in a context of reduced Ca^2+ ^are treated by the nervous system as a single trial; conversely, a single trial in a context of increased Ca^2+ ^mimics the effect of multiple trials. It should be noted that caffeine treatment had to be associated with a conditioning trial, as caffeine injected 20 min before a CS-only presentation did not lead to any LTM performance. Therefore, Ca^2+ ^is sufficient for LTM formation only if an odor-sucrose association has taken place. This suggests that increased [Ca^2+^]i at the moment of a conditioning trial can act as the trigger for biochemical pathways that will lead to *de novo *protein synthesis, and to LTM.

We have found here that the modulation of [Ca^2+^]i during learning affects specifically LTM while leaving learning and short-term memory (STM) intact. Similar results have been observed in rats in which blocking of the L-type voltage-gated Ca^2+ ^channel with the antagonist verapamil also impaired LTM selectively and showed no effect on learning or STM [23]. All these data suggest that a threshold of [Ca^2+^]i level has to be reached during learning to trigger downstream pathways leading to *de novo *gene transcription required for LTM formation. It does not, however, mean that learning and STM do not rely at all on Ca^2+^. In fact, this might indicate that they require different ranges of [Ca^2+^]i than LTM. In any case, the memory formed before 24 h in honeybees is not dependent on *de novo *gene transcription [30].

Our conclusion that Ca^2+ ^may be a memory trigger is further paralleled by an optical imaging study in *Drosophila*, which found increased Ca^2+ ^responses in particular types of mushroom body intrinsic neurons (α'/β' neurons) between 5 and 60 min after a single training cycle [39]. Therefore, our working model is that during acquisition, the first conditioning trial would increase Ca^2+ ^levels in some parts of the olfactory network involved in memory formation, which would then trigger cascades, allowing LTM formation at the subsequent learning trials.

An important point is that Ca^2+ ^alone does not contain the odor specificity for LTM. Indeed, in our experiments, drugs inducing [Ca^2+^]i increase were applied to the whole animal and likely affected all brain structures, yet the enhanced memory was odor specific. This is because odor specificity is ensured by mechanisms within the subset of neurons activated during the single CS-US trial, in particular the neurons that represent the CS in the olfactory network. This could correspond to subsets of antennal lobe neurons (projection neurons and/or local interneurons) or mushroom body neurons (Kenyon cells), which receive input from the US pathway (the ventral unpaired median neuron of the maxillary neuromere 1 (VUM_mx1_) [40]) and are involved in olfactory learning and memory [41,42].

We showed clearly that Ca^2+ ^is critical for LTM. However, we have not determined the origin of Ca^2+^, that is, influx or release from internal stores. Neuropharmacological experiments have shown that nicotinic acetylcholine receptors are involved in the formation of LTM in bees [43]. These receptors could allow the entry of Ca^2+ ^directly through their ion channel or indirectly after depolarization via voltage-gated Ca^2+ ^channels. Moreover, octopamine released by the VUM-mx1 neuron in response to sucrose activates a receptor which can also induce an increase of [Ca^2+^]i [44]. Thus, the convergence of these two Ca^2+ ^influxes in olfactory cells could prime the system for LTM formation. In addition, our caffeine experiments suggest that Ca^2+ ^released from internal stores may also participate in LTM formation. Indeed, caffeine induces the release of Ca^2+ ^from ryanodine-sensitive stores [33] and several behavioral studies have demonstrated that an inhibition of ryanodine receptors impairs spatial [45,46] and passive avoidance memory [47,48]. Other behavioral studies have shown evidence for the role of Ca^2+ ^in the formation of LTM by blocking IP3-receptor-dependent intracellular Ca^2+ ^stores [25].

Our work suggests that the increase of [Ca^2+^]i during the first conditioning trial determines the fate of olfactory cells when the second trial is applied, inducing the cascades leading to LTM. This second trial can take place 10 min or even later after the first one. Disruption of Ca^2+ ^homeostasis over such a long period of time could have detrimental effects on the survival of the cells. Therefore, we favor the hypothesis that an early and transient Ca^2+ ^signal is sufficient to prime olfactory cells for the formation of LTM, and that a relay bridges the gap between associative trials. One possible hypothesis is that Ca^2+^-dependent signaling pathways could be this relay. For instance, the involvement of CaMKII, a Ca^2+^/CaM-activated kinase, in LTM formation has been demonstrated in several studies [49]. The CaMKII has important molecular regulatory features that make it ideally suited for decoding cytosolic Ca^2+ ^signals and translating these signals into the appropriate cellular response via phosphorylation [50]. Its main functional property is the ability for autophosphorylation. This alters the enzyme such that its activity becomes independent of Ca^2+^/CaM binding [50] and can mediate Ca^2+^-induced signaling on a time scale of several minutes, possibly bridging the gap between conditioning trials. Other molecular cascades, such as the NO-cyclic guanine monophosphate (NO-cGMP) or the cAMP-protein kinase A (cAMP-PKA) pathways which have also been shown to be critical for LTM formation [10,51], could play a similar role, since both NO synthase (NOS) and PKA are highly dependent on Ca^2+^. Furthermore, CaMKII and PKA, for example, can also activate the transcription factor CREB [52] which mediates immediate-early gene transcription [53].

Despite the possible role of Ca^2+^-dependent signaling pathways described above, Ca^2+ ^can also invade the nucleus to play a more direct role in gene transcription required for LTM formation [54]. Indeed, Ca^2+ ^can act directly and rapidly on gene expression through the cis-regulatory element, downstream regulatory element which has been involved in stimulation-transcription coupling mechanisms [55].

Multiple trial conditioning, mediated by Ca^2+^-dependent pathways as described above, will lead to *de novo *protein synthesis. As the honeybee genome sequence is available [28] and microarrays of honeybee genes are now accessible (Roy J Carver Biotechnology Center, Illinois, USA), future research will explore Ca^2+^-dependent genes and their involvement in LTM formation.

## Conclusion

Our work shows that Ca^2+ ^is both a necessary and a sufficient signal during conditioning for the formation of protein-dependent LTM. As intracellular Ca^2+ ^increase is one of the earliest events following neuronal activation during learning, our results suggest Ca^2+ ^could be an early trigger of the cellular cascades leading to *de novo *gene expression-dependent LTM formation. We propose that Ca^2+ ^plays the role of a switch between short- and long-term storage of learned information.

## Methods

### Animals

Honeybees (*Apis mellifera ligustica*) were caught at the hive entrance and were placed into standard harnesses. They were then fed with 5 μl of 50% sucrose solution and kept for 3 h before conditioning.

### Classical olfactory conditioning

Olfactory conditioning of the PER [29] was carried out as described elsewhere [56]. Bees were either subjected to a single conditioning trial (4 sec odor CS, 3 sec sucrose US to antennae and proboscis, 1 sec overlap), or to three conditioning trials with 10 min inter-trial intervals. After conditioning, bees were kept in a dark, humid container at room temperature (20 to 25°C) until retrieval test at 3 h, 24 h or 72 h. These retrieval tests were carried out on separate groups of bees to avoid the possible consequences of multiple testing on the same animals. Bees were fed twice a day with a droplet of 50% sucrose solution except on the morning of the retrieval tests. During these tests, bees were exposed in extinction conditions to the CS and to a new odor in order to check whether the formed memory was CS-specific. All experiments were carried out in a balanced fashion with two odors A and B as CS and new odor, respectively. Thus, every experimental day, A was the CS for half the bees, and B for the other half. We chose for A and B pairs of odors that are well differentiated by bees (2-hexanol vs 1-nonanol or 1-hexanol vs 1-nonanol [34]). The US was 50% sucrose.

### Chemicals

The drugs were injected with a volume of 1 μl into the thorax for pharmacological experiments, or into the head hemolymph for uncaging experiments. For Ca^2+^-blocking experiments, the intracellular Ca^2+ ^chelator (BAPTA-AM) (Molecular Probes, OR, USA) at 500 μM was injected 1 h prior to conditioning, corresponding to the necessary time for drug esterification. In a control experiment, the injection was done 1 h after conditioning. For intracellular Ca^2+^-increase experiments, two treatments were done. Caffeine, allowing liberation from intracellular Ca^2+ ^stores via ryanodine receptors, was applied at 20 mM 20 min before conditioning or, for the control experiments, just after or 1 h after conditioning. Another control experiment was carried out by injecting 20 mM caffeine 20 min before a CS-only presentation. In addition, intracellular Ca^2+ ^was released experimentally (see photolysis below) using the caged Ca^2+ ^donor *O*-Nitrophenyl-ethyleneglycol-bis(β-aminoethyl)-N,N,N',N'-tetraacetoxymethyl ester (NP-EGTA-AM; Molecular Probes, OR, USA) at 20 mM, injected 1 h before conditioning. To inhibit transcription, ACT-D at 1.5 mM was injected 3 h after conditioning.

Caffeine was diluted in bee saline (130 mM NaCl, 6 mM KCl, 4 mM MgCl_2_, 5 mM CaCl_2_, 160 mM sucrose, 25 mM glucose and 10 mM HEPES, pH 6.7). Other drugs were diluted in saline with 0.5% dimethylsulfoxyde (DMSO) for NP-EGTA-AM or 0.1% for BAPTA-AM, except for ACT-D which was diluted in phosphate-buffered saline. Control groups received vehicle injections containing the same DMSO concentration as the corresponding experimental groups. For the conditioning, the odors used were 1-hexanol, 2-hexanol (Fluka, Germany) and 1-nonanol (Sigma-Aldrich, France).

For Ca^2+ ^measurements, we used 33 μM calcium green 2 acetoxymethyl ester (2AM) (Molecular Probes, OR, USA), dissolved in bee saline containing 2.5% pluronic acid F-127 (Molecular Probes, OR, USA). All intracellular Ca^2+ ^probes (with AM component) are currently used for maximum efficiency 1 h after application for *in vivo *Ca^2+ ^imaging [57,58] or for *in vitro *experiments [59]. All materials were obtained from Sigma-Aldrich (France) except otherwise stated.

### Flash photolysis of caged compounds

One hour after injection of the caged compound, bees were exposed to ultra violet (UV) light for 1 min and conditioned 5 min afterwards. To carry out UV photolysis, a custom optical system was built, composed of a 100W mercury arc flash lamp, a UV transmitting fused-silica condenser, and a shutter. The location and focusing of the UV spot were adjusted so that light was homogeneous on the whole brain. The optimum uncaging conditions were 800 μW/cm^2^/min at 360 nm, determined with a UV meter (UVP, San Gabriel, California, USA).

### Calcium imaging

*In vivo *Ca^2+ ^imaging recordings with calcium green 2AM were carried out as described elsewhere [58] with some modifications. Fluorescence was recorded for 60 min at a rate of 1 frame every 6 sec, with a 4 × 4 binning and 50 ms integration time. Ten minutes after the start of the experiment, 10 μl of caffeine (20 mM) or saline were applied onto the brain. Relative fluorescence changes were analyzed on three regions of interest corresponding to three olfactory brain structures (antennal lobe, alpha lobe of the mushroom bodies and lateral protocerebral lobe).

### Data analysis

All experiments were performed with two odors A and B in a balanced protocol (see classical olfactory conditioning, above). As no significant effect of the specific odor used as CS (A *versus *B) appeared in any of the groups, data with both odors were pooled. Acquisition performance within each group was tested using Cochran's Q test. Possible differences of acquisition performance between groups were tested with Mann-Whitney U tests. For retrieval tests, we compared within groups the difference of responses between the CS and the new odor with McNemar tests. Differences in CS or new odor responses between groups were assessed using χ^2 ^tests.

As we systematically tested the CS and a new odor, we also compared memory specificity for the CS between groups. We calculated the proportions of individuals responding to the CS and not to the new odor, later termed 'Specific Response proportion' (% SR). These proportions were compared between groups using χ^2 ^tests. To keep a conservative decision threshold, only significant increases of % SR through drug application were considered as memory enhancement.

In Ca^2+ ^imaging experiments, relative fluorescence changes (% ΔF/F0) are represented in 2-min windows (average of 20 frames), with the 2-min window before drug application as reference. The possible difference in Ca^2+ ^levels between caffeine and control groups was tested at each time window using a paired *t*-test.

## Abbreviations

AC: adenylate cyclase; ACT-D: actinomycin-D; BAPTA-AM: 1,2-bis-(o-aminophenoxy)-ethane-N,N,N',N'-tetraacetic acid tetraacetoxymethyl ester; Ca^2+^: calcium; [Ca^2+^]i: intracellular calcium concentration; Ca^2+^/CaMKII: calcium/calmodulin-dependent kinase II; CaM: calmodulin; cAMP: cyclic adenosine monophosphate; CR: conditioned responses; CREB: cyclic adenosine monophosphate responsive element binding protein; CS: conditioned stimulus; DMSO: dimethylsulfoxyde; LTM: long-term memory; NO: nitric oxide; NP-EGTA-AM: *O*-Nitrophenyl-ethyleneglycol-bis(β-aminoethyl)-N,N,N',N'-tetraacetoxymethyl ester; PER: proboscis extension reflex; PKA: protein kinase A; SR: specific responses; US: unconditioned stimulus; UV: ultra violet; VUM_mx1_: ventral unpaired median neuron of the maxillary neuromere 1.

## Authors' contributions

EP carried out most of the experiments, performed the statistical analysis and participated in its design, and contributed to the draft of the manuscript. VR, IN, CL, MM and JCS conceived the study, participated in its design and coordination and helped to draft the manuscript. YM carried out part of the behavioral experiments. JCS helped to perform the statistical analysis. All authors read and approved the final manuscript.

## Supplementary Material

Additional file 1 **Control injections of BAPTA-AM and caffeine after conditioning**. Effect of drug injection on long-term memory (LTM) performance (at 72 h), plotted as the difference (ΔSR) of the percentage of specific response (% individuals responding to the conditioned stimulus and not to the new odor) between treated and control groups. A negative value indicates a memory disruption while a positive value indicates a memory improvement. Thus, bees injected with a Ca^2+ ^chelator, BAPTA-AM 1 h before a three-trial conditioning, show a strong and significant memory disruption (see Figure 1 and main text). A control group injected 1 h after conditioning with BAPTA-AM shows only a weak and non-significant decrease of LTM performance compared with controls (χ^2 ^= 0.49, *P *= 0.48; Control: *n *= 56; BAPTA-AM: *n *= 50). Conversely, bees injected with caffeine, allowing a release of Ca^2+ ^from internal stores, 20 min before a single conditioning trial, show improved LTM performance (see Figure 3 and main text). In Control experiments, when caffeine is injected immediately after the conditioning trial (0 min), a near-significant increase of LTM performance is observed (χ^2 ^= 0.49, *P *= 0.078; Control: *n *= 70; Caffeine: *n *= 80). However, injection 1 h after the conditioning trial does not show any memory increase (χ^2 ^= 0, *P *= 0.98; Control: *n *= 39; Caffeine: *n *= 47) (*: *P *< 0.05, ***: *P *< 0.001, (*): nearly significant, NS: non-significant). Click here for file

Additional file 2 **Control for the conditioned stimulus-unconditioned stimulus association needed for the promnesic caffeine effect**. Retention performances at 72 h, as the percentage of specific response (% SR, % individuals responding to the conditioned stimulus (CS) and not to the new odor), between controls and bees injected with caffeine 20 min, before one conditioning trial (CS + unconditioned stimulus (US)) or a CS-alone trial. The results of the CS + US group were presented in details in Figure 3. In the CS-alone situation, neither control nor caffeine-injected bees show any remarkable long-term memory (LTM) performance (χ^2 ^= 0, *P *= 0.97; Control: *n *= 39; Caffeine: *n *= 40). Caffeine therefore has no effect without a full CS-US conditioning trial. (*: *P *< 0.05, NS: non-significant). Click here for file
