# Supplementary figures and images for: Early calcium increase triggers the formation of olfactory long-term memory in honeybees
Source: BMC Biol. 2009 Jun 16;7:30. doi: 10.1186/1741-7007-7-30 (PMC2713209; doi:10.1186/1741-7007-7-30)

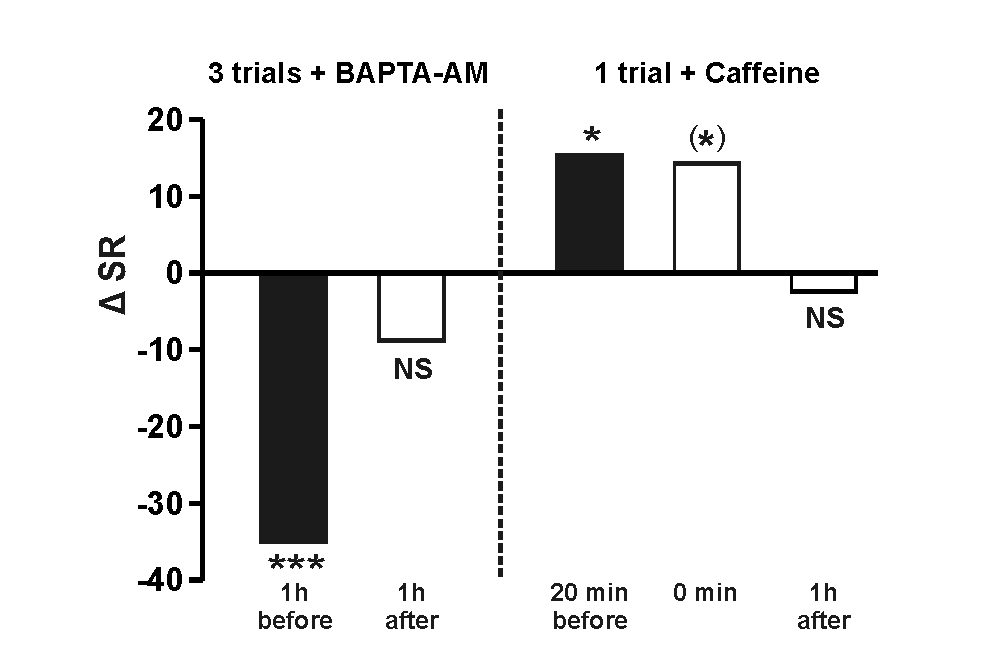

Supplement: Additional file 1 — Control injections of BAPTA-AM and caffeine after conditioning. Effect of drug injection on long-term memory (LTM) performance (at 72 h), plotted as the difference (ΔSR) of the percentage of specific response (% individuals responding to the conditioned stimulus and not to the new odor) between treated and control groups. A negative value indicates a memory disruption while a positive value indicates a memory improvement. Thus, bees injected with a Ca2+ chelator, BAPTA-AM 1 h before a three-trial conditioning, show a strong and significant memory disruption (see Figure 1 and main text). A control group injected 1 h after conditioning with BAPTA-AM shows only a weak and non-significant decrease of LTM performance compared with controls (χ2 = 0.49, P = 0.48; Control: n = 56; BAPTA-AM: n = 50). Conversely, bees injected with caffeine, allowing a release of Ca2+ from internal stores, 20 min before a single conditioning trial, show improved LTM performance (see Figure 3 and main text). In Control experiments, when caffeine is injected immediately after the conditioning trial (0 min), a near-significant increase of LTM performance is observed (χ2 = 0.49, P = 0.078; Control: n = 70; Caffeine: n = 80). However, injection 1 h after the conditioning trial does not show any memory increase (χ2 = 0, P = 0.98; Control: n = 39; Caffeine: n = 47) (*: P < 0.05, ***: P < 0.001, (*): nearly significant, NS: non-significant). [file 1741-7007-7-30-S1.tiff]

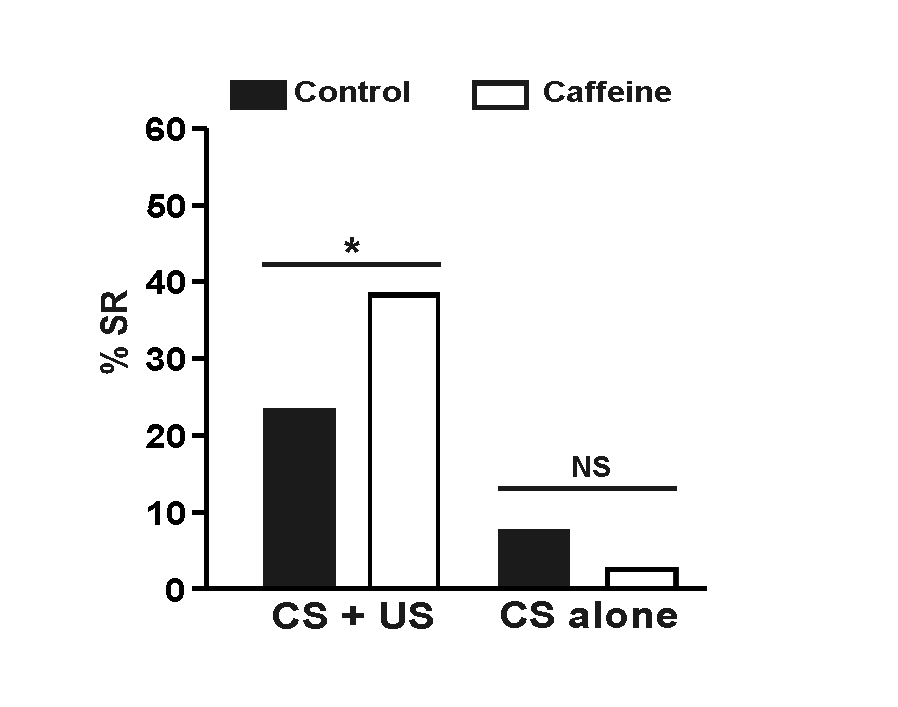

Supplement: Additional file 2 — Control for the conditioned stimulus-unconditioned stimulus association needed for the promnesic caffeine effect. Retention performances at 72 h, as the percentage of specific response (% SR, % individuals responding to the conditioned stimulus (CS) and not to the new odor), between controls and bees injected with caffeine 20 min, before one conditioning trial (CS + unconditioned stimulus (US)) or a CS-alone trial. The results of the CS + US group were presented in details in Figure 3. In the CS-alone situation, neither control nor caffeine-injected bees show any remarkable long-term memory (LTM) performance (χ2 = 0, P = 0.97; Control: n = 39; Caffeine: n = 40). Caffeine therefore has no effect without a full CS-US conditioning trial. (*: P < 0.05, NS: non-significant). [file 1741-7007-7-30-S2.tiff]
